# Bayesian geostatistical modelling of malaria and lymphatic filariasis infections in Uganda: predictors of risk and geographical patterns of co-endemicity

**DOI:** 10.1186/1475-2875-10-298

**Published:** 2011-10-11

**Authors:** Anna-Sofie Stensgaard, Penelope Vounatsou, Ambrose W Onapa, Paul E Simonsen, Erling M Pedersen, Carsten Rahbek, Thomas K Kristensen

**Affiliations:** 1 Center for Macroecology, Evolution and Climate, Department of Biology, University of Copenhagen, Universitetsparken 15, DK-2100 Copenhagen, Denmark; 2 DBL - Centre for Health Research and Development, Faculty of Life Sciences, University of Copenhagen, Thorvaldsensvej 57, DK-1871 Frederiksberg C, Denmark; 3 Department of Public Health and Epidemiology, Swiss Tropical and Public Health Institute, Socinstrasse 57, P.O. Box CH-4002 Basel, Switzerland; 4 Vector Control Division, Ministry of Health, P.O. Box 1661, Kampala, Uganda

## Abstract

**Background:**

In Uganda, malaria and lymphatic filariasis (causative agent *Wuchereria bancrofti*) are transmitted by the same vector species of Anopheles mosquitoes, and thus are likely to share common environmental risk factors and overlap in geographical space. In a comprehensive nationwide survey in 2000-2003 the geographical distribution of *W. bancrofti *was assessed by screening school-aged children for circulating filarial antigens (CFA). Concurrently, blood smears were examined for malaria parasites. In this study, the resultant malariological data are analysed for the first time and the CFA data re-analysed in order to identify risk factors, produce age-stratified prevalence maps for each infection, and to define the geographical patterns of *Plasmodium *sp. and *W. bancrofti *co-endemicity.

**Methods:**

Logistic regression models were fitted separately for *Plasmodium *sp. and *W. bancrofti *within a Bayesian framework. Models contained covariates representing individual-level demographic effects, school-level environmental effects and location-based random effects. Several models were fitted assuming different random effects to allow for spatial structuring and to capture potential non-linearity in the malaria- and filariasis-environment relation. Model-based risk predictions at unobserved locations were obtained via Bayesian predictive distributions for the best fitting models. Maps of predicted hyper-endemic malaria and filariasis were furthermore overlaid in order to define areas of co-endemicity.

**Results:**

*Plasmodium *sp. parasitaemia was found to be highly endemic in most of Uganda, with an overall population adjusted parasitaemia risk of 47.2% in the highest risk age-sex group (boys 5-9 years). High *W. bancrofti *prevalence was predicted for a much more confined area in northern Uganda, with an overall population adjusted infection risk of 7.2% in the highest risk age-group (14-19 year olds). Observed overall prevalence of individual co-infection was 1.1%, and the two infections overlap geographically with an estimated number of 212,975 children aged 5 - 9 years living in hyper-co-endemic transmission areas.

**Conclusions:**

The empirical map of malaria parasitaemia risk for Uganda presented in this paper is the first based on coherent, national survey data, and can serve as a baseline to guide and evaluate the continuous implementation of control activities. Furthermore, geographical areas of overlap with hyper-endemic *W. bancrofti *transmission have been identified to help provide a better informed platform for integrated control.

## Background

Malaria and lymphatic filariasis are two of the most common mosquito-borne parasitic diseases worldwide. Their overall prevalence and health significance have made them top priorities for global control and elimination [1,2]. To plan and evaluate such activities in a cost-effective manner, reliable baseline maps of the geographical distribution of at-risk areas and estimates of the number of infected individuals are important tools to guide and evaluate the continuous implementation of control activities. Likewise, identifying areas of co-endemicity (geographical overlap) is a main operational issue [3] for recently advocated integrated control planning [3-5].

In Uganda, malaria is highly endemic with temperature and rainfall allowing stable, year-round transmission with relatively little seasonal variability in most parts of the country [6]. *Plasmodium falciparum *is by far the most common of the existing malaria species in Uganda, contributing 90-98% of the parasite population [7,8] Despite being a leading cause of morbidity and mortality [9], only relatively few, community-based studies of malaria have been carried out in Uganda [7,10,11]. Malaria risk and endemicity have previously been assessed as part of global-coverage mapping projects based on historical data or climatic suitability [12-16]. However, no detailed empirical risk map has been developed based on coherent national survey data collected in a standardised manner.

Besides malaria, several neglected tropical diseases are reported to be co-endemic in Uganda [11,17] among these bancroftian lymphatic filariasis, resulting from infection with the mosquito-borne parasitic nematode *Wuchereria bancrofti*. Despite being recognized as a major public health and socio-economic problem, knowledge about the occurrence of lymphatic filariasis in Uganda was scanty, until a school-based survey in 2000-2003, allowed the geographical distribution of lymphatic filariasis throughout the country to be mapped [18]. During the same survey, malariological data were collected, but until now no analysis has been presented of these data.

In Uganda, as in many other tropical regions, *Plasmodium *and *W. bancrofti *parasites share common mosquito vector species [19,20]. Thus, they may not occur independently of each other, and the risk of co-infection (multiple species infection) might be considerable. The outcome of parasite co-infections often differs significantly from that of single infections [21,22], and many of the major human infections are known to be affected by the presence of other pathogens altering disease severity, levels of infection or the spatio-temporal disease patterns [23-29]. Yet, only few epidemiological studies explicitly address e.g. malaria-filarial co-infection and/or co-endemicity.

In the present paper, ecological correlates and risk factors of both malaria and lymphatic filariasis infections in Uganda are investigated, and nation-wide risk maps are developed using solid empirical survey data and model-based geostatistics.

The objectives were two-fold: *i*) to derive statistically robust prevalence estimates of malaria and lymphatic filariasis infections separately at un-sampled locations (smooth prevalence maps), and *ii*) to determine the extent of geographical overlap (co-endemicity) of the two parasitic infections. Risk estimates are moreover linked to population data to calculate the number of school-aged children at risk of each infection separately, as well as the number of children living in areas of overlapping hyper-endemic malaria and lymphatic filariasis transmission.

## Methods

### Survey data

**Figure 1:**
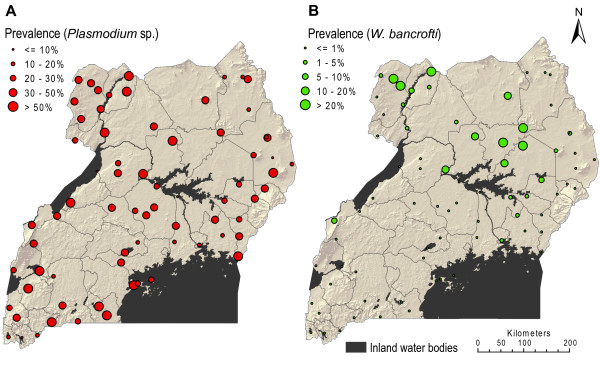
**Maps of survey locations and observed prevalence of infections**. A) *Plasmodium *sp. parasitaemia in 71 schools and B) *Wuchereria bancrofti *antigenemia in 76 schools (children aged 5-19 years) in Uganda, 2000 - 2003.

The screenings were carried out between October 2000 and April 2003 and included pupils aged 5 - 19 years of age from Ugandan primary schools covering all major topographical and ecological zones of the country. Details about survey design and sampling methods have already been given elsewhere [18]. Briefly, a finger-prick blood sample (100-*μ*l) was first collected from each child and examined for *W. bancrofti*-specific circulating filarial antigens (CFA) using commercial immunochromatographic cards. When possible, and when the child agreed another (100- *μ*l) blood sample was collected and used to prepare a thick smear to examine for infection with malaria parasites. Malaria parasites were identified to the genus level, and will hereafter be referred to as *Plasmodium *sp. As not every subject allowed a second sample to be collected, the number of children examined for *Plasmodium *sp. was slightly lower than the number examined for *W. bancrofti *antigens. In total, malariological data was available from 71 schools (total of 11,481 pupils), whereas *W. bancrofti *infection data were available from 76 schools (total of 17,533 pupils) [18,30]. Boys and girls were examined in approximately equal numbers. A map showing the locations of the surveyed schools and the raw prevalence data can be seen in Figure 1.

The studies which contributed data used in this paper, received ethical clearance from the Uganda National Council for Science and Technology and were approved by the Central Scientific Ethical Committee of Denmark.

### Environmental and population density data

Satellite sensor data on Normalized Difference Vegetation Index (NDVI) and day- and night-time land surface temperatures from the Moderate Resolution Imaging Spectroradiometer (MODIS) satellite were obtained from the United States Geological Survey U.S. Geological Survey (USGS) Land Processes Distributed Active Archive Center (LP DAAC) [31] at 1 km resolution. NDVI, is used as a surrogate for moisture availability [32].

The products were downloaded for every 8 - 16 days in the period 2000 to 2003 and average annual composite maps produced by combining all maps in the period 2000-2003 to reduce the variation between climate years. Based on Uganda's two principal rainy seasons, March to May, and August to November, average wet season and dry season composite maps were similarly produced. A climate surface grid at 5 × 5 km resolution consisting of monthly long-term normal rainfall and evapotranspiration estimates was obtained from the IGAD/Nile Minimum Medical Database (MMDb) [33] and seasonal and annual averages calculated. Altitude was determined from a Digital Elevation Model layer produced from NASA's Shuttle Radar Topography Mission (SRTM) [34]. Permanent water bodies, surrounding wetlands and their boundaries were identified through the National Biomass Study [35] and validated using the 2000-2003 MODIS satellite products. A distance grid was developed based on the Euclidean distance between each school and the nearest permanent surface water body.

Environmental data for model fitting were extracted using the satellite positions of each school, as the average of the environmental conditions found within a circular area of 1 km buffered around each school position.

A gridded population surface at 1 km^2 ^resolution was obtained from LandScanTM 2002 Global Population Database [36]. The percentage of boys and girls in each age group (5-9, 10-14 and 15-19 years) out of the total Ugandan population was obtained from the 2002 data of the international data base of the U.S Census Bureau [37].

For the purpose of predicting infection risk at unobserved locations, a grid covering Uganda at 2 × 2 km resolution was constructed, (resulting in approximately 60,000 grid cells) and the average environmental data extracted for each grid cell. Environmental grid data was managed with ArcGIS version 9.3 (ESRI; Redlands, USA) with the Geospatial Modelling Environment (GME) extension [38]

### Statistical analysis

School-children were classified into three age groups of approximately equal sizes (group 1: children aged 5-9 years, group 2: children aged 10-14 years and group 3: children aged 15-19 years) following Onapa et al. [18].

The Pearson χ^2^-test was used to assess associations between sex and age with *Plasmodium *sp. and *W. bancrofti*, single and mono-infections. Single infection refers to infection with a particular species irrespective of other infections that may be present in the individual, whereas mono-infection refers to infection with only the particular species of parasite in question.

Bivariate logistic regression models for each parasitic infection were developed to investigate the relationship between the outcome variable (single infection status) and covariates (demography and environmental themes). To account for clustering at the school-level, a location-specific random effect was included in the bivariate models. To reduce confounding arising from correlated environmental variables, significant variables within the same environmental themes were ranked according to the goodness of fit Akaike Information Criteria (AIC) [39] and the best fitting one selected for further modelling. These analyses were carried out in STATA version 10 (Stata, College Station, TX, USA).

Environmental and demographic covariates with significance level *p *< 0.15 were then built into Bayesian logistic regression models, using the Bayesian statistical software OpenBUGS (vs 3.1.1., Imperial College & Medical Research Council; London, UK) [40]. For each parasite separately, two models with school-level random effects to take geographical dependence into account were fitted : *i*) an exchangeable model assuming that random effects arise from an independent normal distribution with mean 0 and variance quantifying extra binomial variation, and *ii*) a geostatistical model assuming that random effects arise from a Gaussian spatial process quantifying geographical correlation. Model fit was implemented via Monte Carlo methods which allow flexibility in fitting complex models and avoid asymptotic inference and the computational problems encountered in likelihood-based fitting [41]. Furthermore, as exploratory analysis (inspection of scatter plots with a fitted locally weighted scatter-plot curve) indicated non-linearity between infection status and a number of climatic variables, models with categorized climatic variables were also fitted for comparison.

Model-based predictions of prevalence at unobserved locations were obtained via Bayesian predictive distributions for the models [41], using Revolution R Enterprise (vs 4.0, Revolution Analytics). Key outputs are probability distributions of the predicted prevalence at the un-sampled locations that can be summarized by a mean, standard deviation and Bayesian Credible intervals (BCI).

To assess the best performing prediction model, model fit was also carried out on a randomly selected subset (~80%) of the schools (training set). The remaining locations, comprising a simple random sample, were used for validation (test localities). The 95% credible interval of the posterior distribution at the test localities were calculated, and the best performing model for each age group was considered the one with the highest proportion of test locations having observed prevalences within this interval.

A complete mathematical description of the models used is given in the Additional file 1.

## Results

### Parasitological findings

The observed infection status with single- or mono-infections of school-children, stratified by sex and age can be seen in Table 1.

**Table 1:** Comparison of demographic factors associated with *Plasmodium *sp. and *W. bancrofti *single and mono-infections

	***Plasmodium *sp**.				*W. bancrofti*			
	
	# children infected (%)	School prevalence range in %	Χ^2^	*P *- value	# children infected (%)	School prevalence range in %	Χ^2^	*P *- value
**Single infection**								
Sex								
Male	2224 (38.9)	8.1 to 73.4			359 (4.1)	0 to 31.1		
Female	2108 (36.5)	6.7 to 73.3	7.14	0.008	331 (3.7)	0 to 30.5	1.85	0.173
Age groups								
5 to 9	1509 (43.8)	0 to 81.5			139 (2.9)	0 to 25.0		
10 to 14	2417 (35.8)	0 to 76.3			419 (4.0)	0 to 34.4		
15 to 19	401 (31.4)	0 to 53.2	86.6	< 0.001	132 (6.3)	0 to 49.0	44.8	< 0.001
**Mono-infection**								
Sex								
Male	2114 (37.6)	8.1 to 77.6			104 (1.8)	0 to 19.6		
Female	2005 (35.4)	6.7 to 73.7	5.6	0.018	105 (1.9)	0 to 25.0	0	0.978
Age groups								
5 to 9	1453 (43.0)	0 to 81.3			26 (0.8)	0 to 18.7		
10 to 14	2287 (34.4)	0 to 76.1			149 (2.3)	0 to 19.5		
15 to 19	379 (30.6)	0 to 61.9	93.4	< 0.001	34 (2.7)	0 to 23.8	32.6	< 0.001

Overall observed prevalence of single *Plasmodium *sp. infection was 35.2%, ranging from 7.4% in sites in the Southern and North-West of Uganda, to 75.5% on the eastern shore of Lake Victoria (Figure 1). Prevalence was highest in the 5-9 year olds (43.8%), dropping to 31.4% in the oldest age group. Boys were more infected than girls (38.9% versus 36.5%).

Overall observed CFA (*W. bancrofti*) prevalence was 3.9% ranging from 0% in sites in the southern parts in the country to 30.7% in the West Nile area and North of Lake Kyoga. Prevalence was lowest in the 5-9 year olds (2.9%) and peaked in the oldest school-children (6.3%), but there was no significant difference between boys and girls.

**Figure 2:**
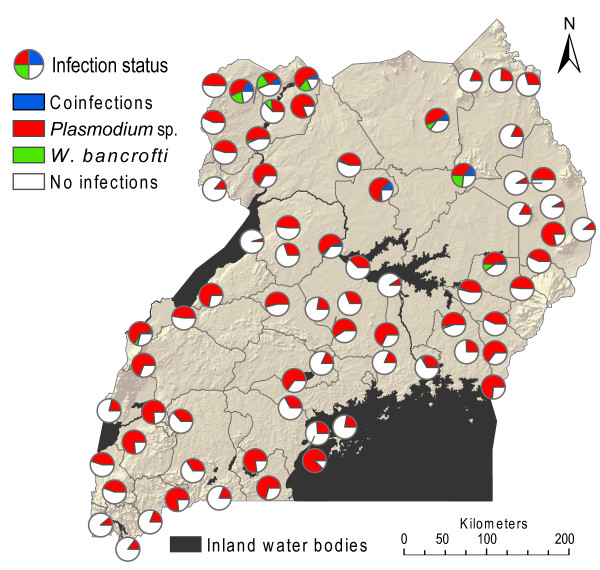
**Observed distribution of mono- and co-infections with *Plasmodium *sp. and *W. bancrofti***. Data from 11,481 pupils aged 5 - 19 years in 71 schools in Uganda, collected during 2000-2003.

The overall prevalence of co-infection was 1.1% (129 individuals out of 11,481 co-infected). In general, more boys than girls had co-infections (72 versus 57 individuals). The age group with the highest level of co-infection was the 10-14 year olds (87 individuals (0.9%)). Figure 2 depicts the geographical distribution of the raw prevalence data of *Plasmodium *sp. and *W. bancrofti *mono- and co-infection, with the school as the unit of analysis. Co-infection was observed in 20 out of 71 schools, with the highest frequencies, exceeding 10%, found in schools in the northern parts of the country.

### Bayesian model validation

The summary results of the model validation can be seen in Table 2, and are presented first since inference and final predictions are based on the best performing models. For malaria both the exchangeable and geostatistical models were able to correctly predict most of the test locations (71 - 93%) within the 95% BCI of the posterior predictive distributions. However, the exchangeable model also produced the overall narrowest BCI widths, and was thus considered the model with the best predictive performance

**Table 2:** Model validation summary for the exchangeable (non-spatial) and geostatistical models of i) malaria and ii) filariasis parasitaemia risk

	Age group	Malaria 95% BCI* (width)	Filariasis 95% BCI* (width)
**Exchangeable models**			
Linear	5-9 yrs	79% (0.60)**	87% (0.32)
	10-14 yrs	86% (0.58)**	87% (0.35)
	15-19 yrs	100% (0.56)**	87% (0.36)
	
Categorical	5-9 yrs	79% (0.64)	87% (0.37)
	10-14 yrs	86% (0.62)	87% (0.42)
	15-19 yrs	100% (0.65)	93% (0.45)

**Geostatistical models**			
Linear	5-9 yrs	71% (0.61)	93% (0.15)**
	10-14 yrs	86% (0.60)	93% (0.21)**
	15-19 yrs	100% (0.58)	93% (0.26)**
	
Categorical	5-9 yrs	71% (0.66)	87% (0.28)
	10-14 yrs	86% (0.65)	87% (0.36)
	15-19 yrs	100% (0.67)	93% (0.35)

For each model, the table shows the percentage of test locations with observed prevalence falling within the 95% Bayesian credible intervals of the posterior predictive distribution and the corresponding BCI width.

Linear and categorical refers to models with co-variates in either linear or categorized form.

** model with the highest percentage of test localities with observed prevalence falling within the 95% BCI and overall narrowest BCI width.

For *W. bancrofti *the geostatistical models were able to predict more test locations correctly within the 95% BCI (87-93%) than the exchangeable models, and also showed the narrowest overall BCIs.

For both malaria and lymphatic filariasis the linear models performed as good as or better than the categorical models, with the linear models predicting as many or more of the test locations correctly, and with overall narrower BCI's for the linear models.

### Predictors of single infections

Associations of malaria parasitaemia and *W. bancrofti *antigenaemia risk with demographic and environmental factors resulting from the bivariate and the Bayesian multivariate exchangeable and geostatistical models with linear terms can be seen in Table 3 and Table 4, respectively. The estimates are presented from the bivariate logistic regression models and Bayesian models with either exchangeable random effects or a spatial location specific random effect. Co-variate effects differed markedly between the two parasitic infections.

**Table 3:** Malaria parasitaemia risk factors as identified from single infection bivariate non-spatial and Bayesian multivariate exchangeable and geostatistical models (*N *= 11,481)

Covariates	Bivariate logistic regression model (non-spatial) OR 95% CI	Bayesian logistic regression model (exchangeable) OR 95% BCI*	Bayesian geostatistical model (spatial) OR 95% BCI*
Age group (5 - 9 yrs)	1.00	1.00	1.00
10 - 14 yrs	**0.67 (0.60 - 0.73)**	**0.66 (0.60 - 0.73)**	**0.66 (0.60 - 0.72)**
15 - 19 yrs	**0.55 (0.47 - 0.63)**	**0.55 (0.46 - 0.64)**	**0.54 (0.47 - 0.64)**

Sex (female)	1.00	1.00	1.00
Male	**1.12 (1.03 - 1.21)**	**1.14 (1.05 - 1.23)**	**1.14 (1.05 - 1.23)**

Season (dry)	1.00	1.00	1.00
Wet	**2.30 (1.54 - 3.44)**	**2.23 (1.43 - 3.23)**	**2.33 (1.50 - 3.53)**

NDVI (wet season)	**1.03 (1.01 - 1.04)**	**1.02 (1.00 - 1.05)**	**1.02 (1.00- 1.05)**
Precipitation (wet season)	**1.01 (1.00 - 1.02)**	1.00 (0.99 - 1.01)	1.00 (0.99 - 1.01)
LST diurnal range (annual)	**0.94 (0.88 - 0.99)**	1.04 (0.95 - 1.14)	1.04 (0.95 - 1.15)

		Mean (95% BCI)	Mean (95% BCI)
τ^2 ^(non-spatial variance)	-	2.00 (1.34 - 2.82)	
σ (spatial variance)	-		0.53 (0.36 - 0.77)
Range (in km)	-		0.08 (0.02 - 0.65)

Results are presented as odds ratios (OR) with their respective confidence intervals (95% CI) or Bayesian credible intervals (95% BCI). NDVI, normalized difference vegetation index; LST, land surface temperature. Range indicates the distance at which spatial correlation is lower than 5%.

**Table 4:** Lymphatic filariasis antigenemia risk factors as identified from single infection bivariate non-spatial and Bayesian multivariate exchangeable and geostatistical models (*N *= 17,533)

Covariates	Bivariate logistic regression model (non-spatial) OR 95% CI	Bayesian logistic regression model (exchangeable) OR 95% BCI*	Bayesian geostatistical model (spatial) OR 95% BCI*
Age group (5 - 9 yrs)	1.00	1.00	1.00
10 - 14 yrs	**2.04 (1.65, 2.55)**	**2.11 (1.69, 2.61)**	**2.13 (1.70, 2.64)**
15 - 19 yrs	**2.47 (1.88, 3.25)**	**2.53 (1.90, 3.31)**	**2.54 (1.91, 3.31)**

Season (dry)	1.00	1.00	1.00
Wet	**3.86 (0.66 - 22.4)**	4.24 (0.55, 13.65)	3.38 (0.99, 7.94)

NDVI (dry season)	**0.93 (0.87, 0.99)**	**0.91 (0.89, 0.94)**	**0.90 (0.86, 0.93)**
Precipitation (annual)	**1.04 (1.00, 1.09)**	**1.06 (1.02, 1.09)**	**1.06 (1.03, 1.09)**
LST (day, dry)	**1.48 (1.17, 1.86)**	1.13 (0.95, 1.36)	1.20 (0.90, 1.22)
LST (night, dry)	**2.56 (1.55, 4.25)**	1.42 (0.95, 2.01)	1.22 (0.83, 1.79)

Altitude (< 1.050 m)			
1.050 - 1.150	0.26 (0.04, 1.71)	0.66 (0.06, 2.81)	**0.15 (0.01, 0.71)**
1.150-1.250	**0.06 (0.01, 0.49)**	**0.17 (0.01, 0.84)**	0.28 (0.02, 1.82)
> 1.250	**0.01 (0.00, 0.14)**	**0.15 (0.03, 0.76)**	**0.21 (0.02, 0.93)**

		Mean (95% BCI)	Mean (95% BCI)
τ^2 ^(non-spatial variance)	-	0.15 (0.08, 0.26)	-
σ (spatial variance)	-	-	4.34 (2.25, 8.80)
Range (in km)	-	-	2.98 (1.78, 4.29)

Results are presented as odds ratios (OR) with their respective confidence intervals (95% CI) or Bayesian credible intervals (95% BCI); NDVI, normalized difference vegetation index; LST, land surface temperature. Range indicates the distance at which spatial correlation is lower than 5%.

For the *Plasmodium *sp., the non-spatial bivariate logistic regression analysis and likelihood ratio tests showed that age, sex, season of data collections and all remotely sensed factors were significant at the 15% significance level. Males and younger children had a significantly higher risk of parasitaemia than females and older children. Diurnal temperature range and precipitation and NDVI composited over the wet season performed better than the other seasonal composites as measured by AIC (results not shown), and was thus selected for inclusion in Bayesian multivariate models. However, only the positive relationship with NDVI remained significant in these models.

The non-spatial bivariate analysis of *W. bancrofti *infection revealed that age, season, elevation and all remotely sensed environmental factors were significant risk factors. High risk was related to high average annual rainfall and low dry season NDVI. The positive associations with day- and night-time temperatures and season were no longer significant in the Bayesian multivariate models. Older children had a significantly higher risk of being CFA positive, whereas sex was not significant at the 15% significance level and thus not included in the Bayesian models. The spatial decay parameter in the geostatistical model had a posterior mean of 1.00 which in the current exponential setting corresponds to a minimum distance for which the spatial correlation becomes (less than 5%) of 3 km (95% BCI: 1.78, 4.29 km)), indicating the presence of spatial autocorrelation in the filariasis data.

### Prediction of *Plasmodium *sp. and *W. bancrofti *single infections

**Figure 3:**
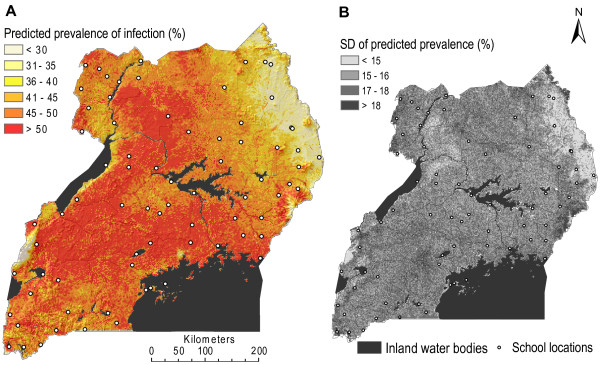
**Map of predicted malaria prevalence**. A) Predicted *Plasmodium *sp. parasitaemia risk for schoolchildren in the highest risk group (5-9 years old boys) and B) associated map of the standard deviation of the predicted risk.

**Figure 4:**
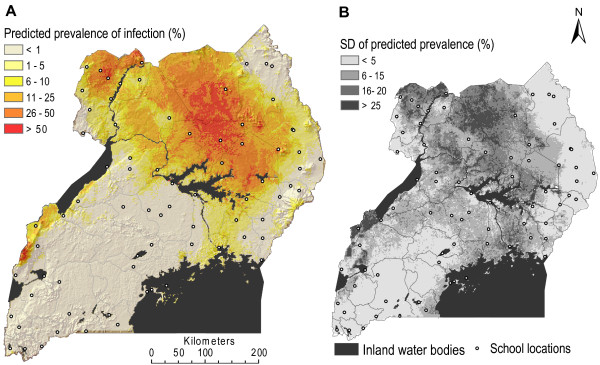
**Map of predicted lymphatic filariasis prevalence**. A) shows predicted *W. bancrofti *antigenemia risk for school-children in the highest risk age group (14-19 years) and B) shows the associated map of the standard deviation of the predicted risk.

The best performing models according to the model validation procedure were employed to predict infection risk at the un-sampled locations represented by the app. 60,000 grid cells across Uganda. The predicted risk maps of single malaria and lymphatic filariasis infections are shown in Figure 3 and Figure 4, respectively.

Since age remained a significant risk factor for both malaria and lymphatic filariasis after adjusting for confounding factors and spatial correlation, prediction maps were developed for each age group separately. For malaria this was also the case for gender. The spatial patterns were identical for each age-sex group, though the proportion infected with *Plasmodium *sp. was lower for girls and older children, and the proportion infected with *W. bancrofti *lower for younger children. Risk maps for all age-sex groups are available from the corresponding author upon request.

### Estimating the numbers infected

**Figure 5:**
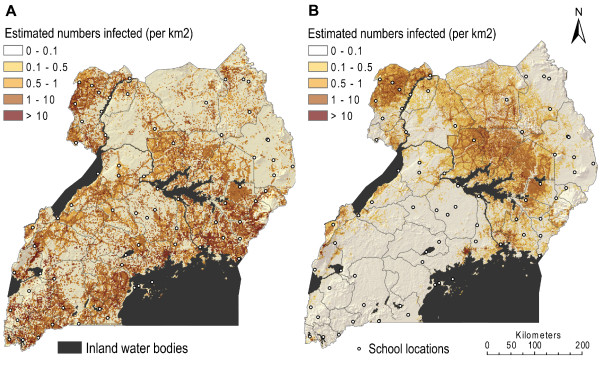
**Maps of the estimated at-risk populations in Uganda, 2002**. The maps show the estimated numbers of school-children aged 5-19 years per km^2 ^infected with *Plasmodium *sp. parasites (A) and *W. bancrofti *filarial parasites (B)

The age and sex stratified 2002 population surfaces was multiplied with gridded surfaces of the prediction models representing the mean and the lower and upper 95% BCI of the predicted prevalence in each age-sex group, for each parasite infection separately. The resulting layers gave maps of the predicted number of infected children per km^2 ^and summarizing across the whole study area gave the total number of infected children in each age-sex group in Uganda. The estimated number of children at risk in the respective age-sex groups is presented in Table 5, along with the population adjusted prevalence for each age-sex group. The total predicted number of school-children per km^2 ^with either malaria or filarial parasites is illustrated in Figure 5A and Figure 5B, respectively.

**Table 5:** Estimated numbers of children infected with a) *Plasmodium *sp. and b) *W. bancrofti *filarial parasites in Uganda in 2002

Age-sex group	Estimated population	Estimated number of infected children	Lower 95% BCI*	Upper 95% BCI*	Model based prevalence	Model-based population adjusted prevalence
**a) *Plasmodium *sp**.						
Boys 5-9 yrs	1,966,324	927,634	337,740	1,547,775	47.7% (4.5)	47.2%
Boys 10-14 yrs	1,587,915	607,356	190,873	1,128,372	38.8% (4.3)	38.2%
Boys 14-19 yrs	1,268,729	431,452	125,464	848,679	34.6% (4.1)	34.0%
Girls 5-9 yrs	1,956,764	607,291	163,873	1,252,415	31.5% (3.5)	31.0%
Girls 10-14 yrs	1,576,155	372,161	90,670	852,659	24.1% (3.3)	23.6%
Girls 14-19 yrs	1,247,670	254,625	55,329	614,181	21.0% (3.0)	20.4%

**TOTAL**	**9,603,557**	**3,200,519**	**963,949**	**6,244,081**	**33.0%**	**33.3%**

**b) *W. bancrofti***						
5-9 yrs	3,923,088	170,668	5,027	691,754	6.7%	4.4%
10-14 yrs	3,164,070	202,885	8,416	733,247	9.3%	6.4%
14-19 yrs	2,516,399	180,860	7,893	665,842	10.1%	7.2%

**TOTAL**	**9,603,557**	**554,413**	**21,336**	**2,090,843**	**8.7%**	**6.0%**

* Bayesian credible interval

### Co-endemicity mapping

**Figure 6:**
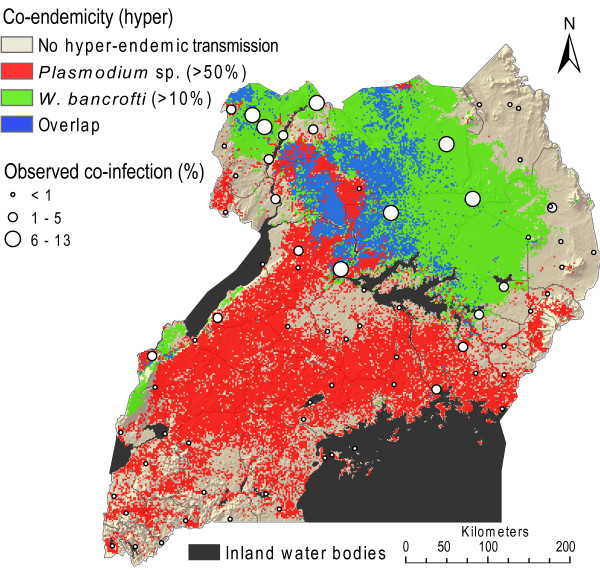
**Map showing areas of hyper-endemic malaria (red) and *W. bancrofti *infections (green) and their geographical overlap (blue) for children aged 5-9 years in Uganda**. Hyper-endemicity is defined as >50% prevalence of *Plasmodium *sp. infection and >10% *W. bancrofti *infection prevalence.

The predicted prevalence maps for malaria and *W. bancrofti *single-infections were superimposed to identify geographical areas where hyper-endemic malaria (> 50%) and hyper-endemic lymphatic filariasis (> 10%) overlap in Uganda. Figure 6 shows the resultant co-endemicity map for children aged 5 - 9 years. The largest areas of hyper co-endemicity were located in Arua district in northwestern Uganda, Kitgum District in northern Uganda and more scattered areas found on the northern side of Lake Kyoga, central Uganda. Based on this map 212,975 children aged 5 - 9 years were estimated to be living in areas of concomitant hyper-endemic malaria and lymphatic filariasis in Uganda, and thus at risk of co-infection.

## Discussion

With recent advances in geospatial tools and spatial statistics, studies analysing the relationship between infection dynamics and ecological factors have given new insights into the ecology and epidemiology especially of malaria [13,15,42] - but also lymphatic filariasis [43,44]. The accurate geographical identification and enumeration of individuals at risk of single-species infection, the outcome of such studies, is an important component in control efforts, facilitating a better resource allocation, health management and targeted interventions to achieve the highest risk reduction for the most populated areas.

In the present study, nationally representative survey data of malaria and lymphatic filariasis in Uganda were applied to identify significant predictors associated with parasitaemia and antigenaemia risk. Model-based infection risk maps for 2002 were produced and the number of infected children estimated for three different age groups.

The map of malaria is the first for Uganda based on empirical data sampled in a standardised and coherent manner. The survey locations are randomly chosen and the data are available at individual level allowing for estimation of age-specific risk, as well as adjusting for seasonality. Even though the data were collected during 2000-2003, the maps produced still represent the most up-to-date and detailed maps of malaria and lymphatic filariasis risk in the country. On-going LF and malaria control initiatives in Uganda are likely to have changed the patterns, thus the maps can serve as an important baseline to guide and evaluate the continuous control implementation.

Prior to the present analysis, historical malariological data were compiled by the MAP (Malaria Atlas Project) [45] and MARA (mapping malaria risk in Africa) projects and used to prepare parasitaemia risk maps at high resolution. For example, Craig *et al *[12] developed a climatic suitability malaria risk map for the whole of Africa, and the first empirical based map for Uganda was produced by Hay *et al *[13] as part of a global risk map based on historical data to define the spatial limits of malaria within all endemic countries. However, often historical survey data are not representative as high risk areas tend to be over-represented, and surveys at different locations are conducted with different methodology, making direct comparisons between maps difficult.

The first risk map of lymphatic filariasis in Uganda was produced by Onapa *et al *[18] based on the same survey data as used the current study. In this study, it was estimated that 8.7 million people (all age groups) lived in areas with > 1% CFA prevalence, and 5.9 million lived in areas with more than 5% CFA prevalence [18]. Here, this mapping is improved upon by using model-based geostatistical analysis to predict for each pixel in the map the risk of having CFA stratified according to age as well as producing a map of the associated prediction errors. Bayesian inference and MCMC simulation is applied as this is the only way these highly parameterized spatial models can be estimated [46]. Furthermore, the modelling approach applied in the current study allowed estimation of the actual number of school-aged children in each age group expected to be CFA positive. However, as the data was collected over a three-year period (2000-2003), the estimated numbers of infected is likely to diverge from 2002 real numbers.

Bayesian geostatistical modelling has previously been applied to lymphatic filariasis data in Haiti [47,48], West Africa [28] and Papua New Guinea [49], however these studies did not aim to predict risk estimates and produce smooth maps of disease risk.

More commonly, Bayesian geostatistical modelling has been applied to malaria data and used to estimate parasitaemia risk for a number of countries and regions in Africa, including amongst others West and Central Africa [50-52], Angola [53], Tanzania [54], Kenya [55], Zambia [56] and Somalia [57].

An important advantage of Bayesian geostatistical analysis is that it takes the spatial correlation often present in many epidemiological data sets into account and model the effects of the co-variates and spatial correlation, if present, simultaneously. If spatial correlation is present the independence assumption central to generalized linear model theory is violated, which could potentially lead to imprecise risk estimates, prediction errors and wrong estimation of the significance of the risk factors.

The geostatistical model of *W. bancrofti *infection estimated a spatial correlation with a minimum distance at which spatial correlation becomes negligible of ~ 3 km. In accordance with this the spatial models also outperformed the non-spatial LF models in terms of number of test locations captured within the 95% posterior credible intervals and their widths. In line with our findings, Alexander *et al *[58] found that the spatial correlation of *W. bancrofti *in Papua New Guinea was reduced by half over a distance of 1.7 km and Boyd et al found that the distance beyond which remaining spatial correlation is assumed to be negligible to be 2.15 km for *W. bancrofti *in school-children in Leogane, Haiti [59].

The malaria data analysed in the present study on the other hand, showed signs of very weak spatial correlation. Thus attempting to model the spatial correlation could lead to increased number of model parameters while decreasing the precision of the estimates. The Bayesian geostatistical model developed estimated a lower spatial correlation than the pixel size used for prediction (2 × 2 km) indicating that spatial correlation is only present at very local scales. Validation confirmed that the fitted non-spatial malaria models were superior to the fitted spatial models. A similar result was found by Riedel *et al *[56] when mapping malaria risk using the Zambia national indicator survey data. Other studies also reported very small scale of spatial variation of malaria [58,60,61]. For example, Thompson *et al *found the risk of malaria varying by a factor of 6 over 500 m [62], while Alexander *et al *found distance over which the correlation reduces by half to be only 14 meters [58].

The much smaller scale of variation of malaria could be related to its rapid variation in infection over time. One infective mosquito bite results in patent infection about two weeks later. By comparison, the patent period is about one year for *W. bancrofti *infections, which also last much longer and the risk of infection per mosquito bite is much smaller [63].

Besides from mapping, important common environmental risk factors for malarial and filarial infections in Uganda have been highlighted. For both malaria and lymphatic filariasis, the bivariate analysis indicated that the season the data was collected in was important with the wet season months having higher disease risk. However, in the multivariate models this relationship only remained significant for malaria reflecting the stronger seasonality of malaria transmission.

As also presented by Onapa *et al *[18] older age groups were significantly more at risk of being CFA positive. The opposite relationship was observed for *Plasmodium *sp. infections, with the youngest age-group being significantly more at risk. This was also the findings of Pullan *et al *[7] in a recent survey of malaria and its risk factors in four villages in Tororo district, Eastern Uganda. Similar to our findings, they also found that boys were significantly more at risk than girls.

### Co-endemicity of malaria and lymphatic filariasis

In contrast to the well-characterized patterns of single parasite infections, we know surprisingly little about the patterns and risk factors of co-endemicity and co-infection of e.g. malaria and helminth infections, despite poly-parasitism being common among populations in developing countries [64]. Co-endemicity is of considerable public health importance and offers opportunities to enhance cost-effectiveness through combined control [3]. The identification and enumeration of individuals living in areas where multiple diseases co-exist is emphasised, since they are at high risk of co-infections, and thus likely to suffer from co-morbidity [65].

In Uganda, the same species of Anopheles mosquitoes transmit malaria and lymphatic filariasis [18,30] and concomitant infections in humans are likely to occur when the prevalence of both parasites is high [19]. In the present study, maps of single infection risk for malaria and lymphatic filariasis were used to identify geographical areas of overlapping hyper-endemic transmission.

A substantial geographical overlap was observed, with an estimated 212,975 children between five and nine years of age living in areas that are hyper-endemic for malaria *and *lymphatic filariasis (blue area in Figure 6). Future surveys should aim at covering these presently un-sampled areas, since the risk of co-infection here would be expected to be substantial.

The geographical distribution of lymphatic filariasis in Uganda was expected to overlap with that of malaria, in particular in very high malaria transmission areas where the density of the transmitting mosquitoes is expected to be high. Surprisingly however, the distribution of *W. bancrofti *antigenaemia is not fully overlapping with hyper-endemic malaria areas (Figure 6), despite most of Uganda being highly endemic for malaria,

A possible explanation for this pattern could be that in some locations, even though data were collected during wet season months, those years and locations were found to be exceptionally dry for the season. This could have impacted the observed prevalence of malaria resulting in lower than usual number of infections; whereas lymphatic filariasis with its much longer prepatent period would remain unchanged even in a dry year. It could also be partly due to the difference in "high prevalence age groups" between the two infections, with far the majority of CFA positives found in age group 15-19 years, the age group with the lowest observed malaria infection.

Some studies have also shown that the intensity of *P. falciparum *is generally lower in microfilaremic individuals compared to amicrofilaraemic individuals [66] and that filarial infections may have either benign or suppressive effects on malaria development [67]. Thus, the two parasites might interact both within the human populations and the vector in ways that may alter the dynamics of transmission [19,68] and ultimately the geographical patterns of co-endemicity.

## Conclusions

The age-sex stratified risk maps of malaria and lymphatic filariasis presented here for Uganda are based on national, coherent survey data collected during 2000-2003 and rigorous Bayesian geostatistical modelling and prediction. They can serve as an important baseline to guide and evaluate the continuous implementation of single infection control activities. The maps were furthermore used to identify areas of hyper- and co-endemicity to help provide a better informed platform for integrated control.

Parasitic co-infection and interaction phenomena in human populations are complex and our understanding of the mechanisms by which interactions between parasites occur and the public health implications is still limited. Further studies on malaria-filarial co-infection, co-occurrence, prevalence and species interactions in various geographical settings are clearly warranted. Besides the immediate public health implications, identifying high-prevalence zones of human parasitic infections and their spatial overlap may also help generate new insights and hypotheses regarding their interrelationships and transmission.

## List of abbrevations

LF: Lymphatic filariasis; CFA: circulating filarial antigens; NDVI: Normalized difference vegetation index; MCMC: Markov Chain Monte Carlo; LST: land surface temperature; BCI: Bayesian credible interval.

## Competing interests

The authors declare that they have no competing interests.

## Authors' contributions

AS and AWO conceived the idea for the present study. AS analysed the data and drafted the manuscript. PV was responsible for the conception and design of the statistical analysis and supervised the implementation. AWO, PES and EMP designed and carried out the parasitological surveys and provided important intellectual content to the study. CR and TKK gave critical input and re-appraisal in the manuscript. All authors read and approved the final manuscript.

## Supplementary Material

Additional file 1 **Additional information regarding model formulation**. Supporting information on Bayesian model formulation for malaria and lymphatic filariasis, Click here for file
