# Supplementary material for: Bayesian geostatistical modelling of malaria and lymphatic filariasis infections in Uganda: predictors of risk and geographical patterns of co-endemicity
Source: Malar J. 2011 Oct 11;10:298. doi: 10.1186/1475-2875-10-298 (PMC3216645; doi:10.1186/1475-2875-10-298)
Supplement: Additional file 1 — Additional information regarding model formulation. Supporting information on Bayesian model formulation for malaria and lymphatic filariasis, [file 1475-2875-10-298-S1.DOCX]

**Supplementary material**

Let *Y_ij_* be the binary outcome of infection status and *p_ij_* the probability of parasitic infection of child *j* at location *i*. We assume that *Y_ij_* arises from a Bernoulli distribution, *Y_ij_* ~ *Be*(*p_ij_*), and model covariates **X***_ij_* = (X*_ij_*_1_,... X*_ij_*_n_)^T^ on the log*it* (*p_ij_*), that is

$$\text{log}\text{it}\text{ }\text{(}\text{p}\text{ij}\text{)}\text{ }\text{=}\text{ }\boldsymbol{X}_{\boldsymbol{i}j}^{T}\text{ }\text{β}$$

where **β** is the vector of the regression coefficients.

To account for unexplained non-spatial variation, a set of location-specific error terms **ε** = (ε_1,...,_ ε_n_) ^T^ were included in the model. They are assumed to be independent, arising from a normal distribution *ε_1_* ~ *N*(0,*τ*^2^) where *τ*^2^ accounts for the non-spatial variation in the infection risk data (exchangeable random effects). The model can thus be written as:

$\text{log}\text{it}\text{ }\text{(}\text{P}\text{ij}\text{)}\text{ }\text{=}\text{ }\boldsymbol{X}_{\boldsymbol{i}j}^{T}\text{ }\text{β}\text{ +}\text{ }\text{ε}$*_i_*

In the geostatistical model, the spatial correlation is introduced as a random effect (error term) φ*_i_* at each location by assuming that **φ** = (φ_1_ ,...φ_n_)^T^ has a multivariate normal distribution, **φ** ~ MVN(0, Σ), with variance-covariance matrix Σ. Moreover, an isotropic spatial process was assumed, i.e., Σ_mn_ = *σ*^2^ exp(-*ρd*_mn_), where d_mn_ is the Euclidean distance between location m and location n, *σ*^2^ is the spatial variation (known as the sill), and *ρ* is a smoothing parameter controlling the rate of correlation decay with increasing distance. For the exponential correlation function, the minimum distance at which the spatial correlation between locations is less than 5% (range of spatial process) is calculated by 3/*ρ*.

This model is written as

$\text{log}\text{it }\text{(}\text{P}\text{ij}\text{)}\text{ }\text{=}\text{ }\boldsymbol{X}_{ij}^{T}\text{ }\text{β}\text{ + }\text{φ}$*_i_*

To complete Bayesian model specification, we assumed independent normal prior distributions for the regression coefficients, with mean 0 and variance 100. For *σ*^2^, *τ*^2^ and *ρ* inverse gamma distributions with mean 1 and variance equal to 100 were adopted. The prior distribution for the *ρ* parameter was furthermore bounded between 700m and 208km based on the minimum and maximum distances between the survey locations and a cutoff correlation of 0.05. Due to the large number of model parameters, Markov chain Monte Carlo simulation methods (Gibbs sampling) were used for model fitting. We ran a single chain sampler with a burn-in of 5000 iterations, followed by 100,000 iterations. Convergence was assessed by inspection of ergodic averages of selected model parameters and convergence was successfully achieved before the 100,000 iterations. After convergence, the algorithm was run for another 25000 iterations to collect a sample of size 500 (1 sample for every 50 iterations).
